# Automatic sleep stage classification based on subcutaneous EEG in patients with epilepsy

**DOI:** 10.1186/s12938-019-0725-3

**Published:** 2019-10-30

**Authors:** Sirin W. Gangstad, Kaare B. Mikkelsen, Preben Kidmose, Yousef R. Tabar, Sigge Weisdorf, Maja H. Lauritzen, Martin C. Hemmsen, Lars K. Hansen, Troels W. Kjaer, Jonas Duun-Henriksen

**Affiliations:** 1 Department of Applied Mathematics and Computer Science, Technical University of Denmark, Richard Petersens Plads, Bygning 324, 2800 Kgs. Lyngby, Denmark; 2 UNEEG medical A/S, Nymoellevej 6, 3540 Lynge, Denmark; 3 Department of Engineering, Aarhus University, Finlandsgade 22, 8200 Aarhus N, Denmark; 4 Center of Neurophysiology, Department of Neurology, Zealand University Hospital, Vestermarksvej 11, 4000 Roskilde, Denmark; 5 Department of Basic and Clinical Neuroscience, King’s College London, 5 Cutcombe Road, SE5 9RX London, UK

**Keywords:** Subcutaneous EEG, Wearable EEG, Automatic sleep scoring, Sleep, Epilepsy

## Abstract

**Background:**

The interplay between sleep structure and seizure probability has previously been studied using electroencephalography (EEG). Combining sleep assessment and detection of epileptic activity in ultralong-term EEG could potentially optimize seizure treatment and sleep quality of patients with epilepsy. However, the current gold standard polysomnography (PSG) limits sleep recording to a few nights. A novel subcutaneous device was developed to record ultralong-term EEG, and has been shown to measure events of clinical relevance for patients with epilepsy. We investigated whether subcutaneous EEG recordings can also be used to automatically assess the sleep architecture of epilepsy patients.

**Method:**

Four adult inpatients with probable or definite temporal lobe epilepsy were monitored simultaneously with long-term video scalp EEG (LTV EEG) and subcutaneous EEG. In total, 11 nights with concurrent recordings were obtained. The sleep EEG in the two modalities was scored independently by a trained expert according to the American Academy of Sleep Medicine (AASM) rules. By using the sleep stage labels from the LTV EEG as ground truth, an automatic sleep stage classifier based on 30 descriptive features computed from the subcutaneous EEG was trained and tested.

**Results:**

An average Cohen’s kappa of $\kappa = 0.78\pm 0.02$ was achieved using patient specific leave-one-night-out cross validation. When merging all sleep stages into a single class and thereby evaluating an awake–sleep classifier, we achieved a sensitivity of 94.8% and a specificity of 96.6%. Compared to manually labeled video-EEG, the model underestimated total sleep time and sleep efficiency by 8.6 and 1.8 min, respectively, and overestimated wakefulness after sleep onset by 13.6 min.

**Conclusion:**

This proof-of-concept study shows that it is possible to automatically sleep score patients with epilepsy based on two-channel subcutaneous EEG. The results are comparable with the methods currently used in clinical practice. In contrast to comparable studies with wearable EEG devices, several nights were recorded per patient, allowing for the training of patient specific algorithms that can account for the individual brain dynamics of each patient. Clinical trial registered at ClinicalTrial.gov on 19 October 2016 (ID:NCT02946151).

## Background

The polysomnography (PSG) is the gold standard to assess sleep stages and other clinically relevant sleep parameters. However, it is resource demanding, impractical for the patient and may in itself have a negative impact on the sleep due to the obtrusive nature of the equipment. This method often limits the sleep assessment to a few days. To gain an objective measurement of sleep patterns over longer periods of time, a variety of wearable sleep trackers have emerged in the recent years. Activity-based devices monitor movements to infer information about sleep–wake patterns, and is currently the modality of choice for long-term sleep monitoring [1, 2]. A review by Sadeh et al. [3] concludes that actigraphy is reliable in individuals with normal sleep patterns. However, the authors question the validity in patients with sleep disorders, poor sleep and certain special populations such as very young children or the elderly. One of the major limitations of the actigraphy that is highlighted is the low specificity reported in several studies (the ability to recognize wakefulness, which affects estimates of, for example, wakefulness after sleep onset and sleep efficiency). In patients with epilepsy, seizures can produce movement patterns that can affect the actigraphy scoring. Sadaka et al. [4] compared actigraphy with continuous video-EEG over a 24-h period in 27 children with medically refractory epilepsy. The authors found that actigraphy reliably estimated commonly used sleep measures except number of wakings after sleep onset. They conclude that actigraphy can be used as a reliable tool for evaluating sleep patterns in children with epilepsy, but as reported in other studies, detecting wake periods after sleep onset remains a challenge.

As the American Academy of Sleep Medicine (AASM) manual mainly differentiates between the stages of sleep based on EEG features, the EEG is an essential tool in sleep monitoring. With the availability of publicly open EEG databases for benchmarking, several studies have developed algorithms for automatic sleep stage scoring of scalp EEG [5–9]. For a review of state-of-the-art feature extraction and classification techniques, see [10]. Other studies have sleep scored EEG from wearable devices such as ear plugs [11, 12], around-the-ear electrode arrays [13], head bands [14] and disposable forehead electrode arrays [15]. Most of these studies report promising results. However, some of the wearables are more suitable for ultralong-term recordings than others.

An emerging EEG modality is subcutaneous EEG. In contrast to currently available wearable EEG solutions, the electrodes are situated in a protected position underneath the skin and can provide continuous measurements with consistent location and impedance for months. Subcutaneous EEG solutions are therefore well suited for ultralong-term monitoring, meaning continuous recordings for > 2 weeks. If wearing a recording device in everyday life is well tolerated by the user, it is believed that long-term monitoring could provide a great help in treatment optimization and alarming of caregivers of patients with epilepsy [16]. It has already been shown that recordings from subcutaneous channels were comparable to those of scalp channels at similar locations [2, 17], and that subcutaneous EEG could be used to detect clinically relevant events in epilepsy patients [2]. The current study shows that subcutaneous recordings can be used to sleep score the same patient population to produce clinically relevant sleep measures. Subcutaneous EEG and LTV EEG from four inpatients were independently sleep scored by a trained expert. By using the sleep stage labels from the LTV EEG as ground truth, an automatic sleep stage classifier based on the subcutaneous EEG was trained and tested. The algorithm was cross-validated (CV) using two strategies: a patient-specific (PS) approach and a leave-one-night-out (LONO) approach. In addition to evaluating the algorithms and the human expert on the five-class sleep staging problem, the hypnograms were converted to sleep–wake traces to create a simpler two-class classification task. Furthermore, some common sleep measures computed from the ground truth hypnograms and the predicted hypnograms were compared.

## Results

### Sleep stage classification

**Fig. 1:**
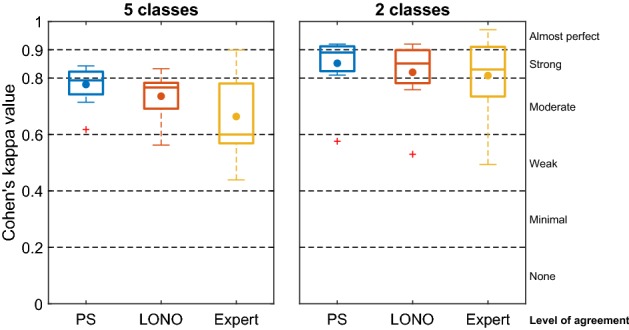
Box plot of Cohen’s kappa values. The midline in the boxes represent the medians, and the dots represent the means. Red crosses are outliers. The mean value ± standard deviation of the mean for the five-class problem: $\kappa _{PS.}=0.78 \pm 0.02$, $\kappa _{LONO}=0.74 \pm 0.02$ and $\kappa _{expert}=0.66 \pm 0.04$. Mean value ± standard deviation of the mean for the two-class problem: $\kappa _{PS.}=0.85 \pm 0.03$, $\kappa _{LONO}=0.82 \pm 0.03$ and $\kappa _{expert}=0.81 \pm 0.04$. The horizontal lines represent intervals of the level of agreement as interpreted by McHugh et al. [18]

**Fig. 2:**
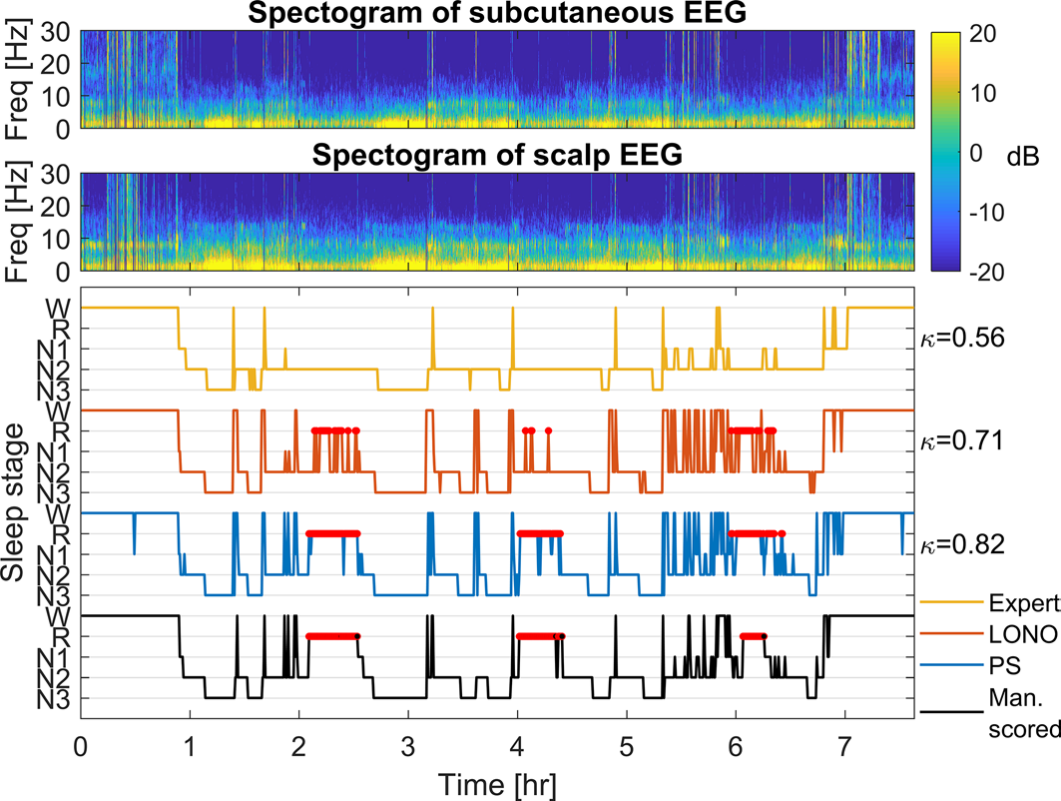
Representative night: the second night of patient B. Top panel: spectrogram of the proximal subcutaneous EEG channel (P–C). Middle panel: spectrogram of the corresponding scalp channel (P7–T7). Bottom panel: manually scored hypnogram based on scalp EEG and the predicted hypnograms by the PS and LONO algorithm and the human expert

The Cohen’s kappa values for the algorithms and the human expert when scoring five and two classes are plotted in Fig. 1 and tabulated in Appendix C. The CV strategy producing the best average Cohen’s kappa value across nights was the PS approach. The mean kappa value ± standard deviation of the mean is $\kappa _{PS.}=0.78 \pm 0.02$, while the mean kappa value for the LONO approach is $\kappa _{LONO}=0.74 \pm 0.02$. For comparison, the mean kappa value for the human expert evaluating the subcutaneous EEG is $\kappa _{expert}=0.66 \pm 0.04$. An exact paired permutation test revealed that the mean kappa values for both algorithms were significantly higher than for the human expert ($p_{PS}=0.0016$, $p_{LONO}=0.015$). For an illustration of a representative night showing the manually labeled and predicted hypnograms, see Fig. 2. For the two-class problem, the PS models had a mean kappa value of $\kappa _{PS}=0.85 \pm 0.03$, the LONO-approach had a kappa of $\kappa _{LONO}=0.82 \pm 0.03$, and the human expert had a kappa of $\kappa _{expert}=0.81 \pm 0.04$.

**Fig. 3:**
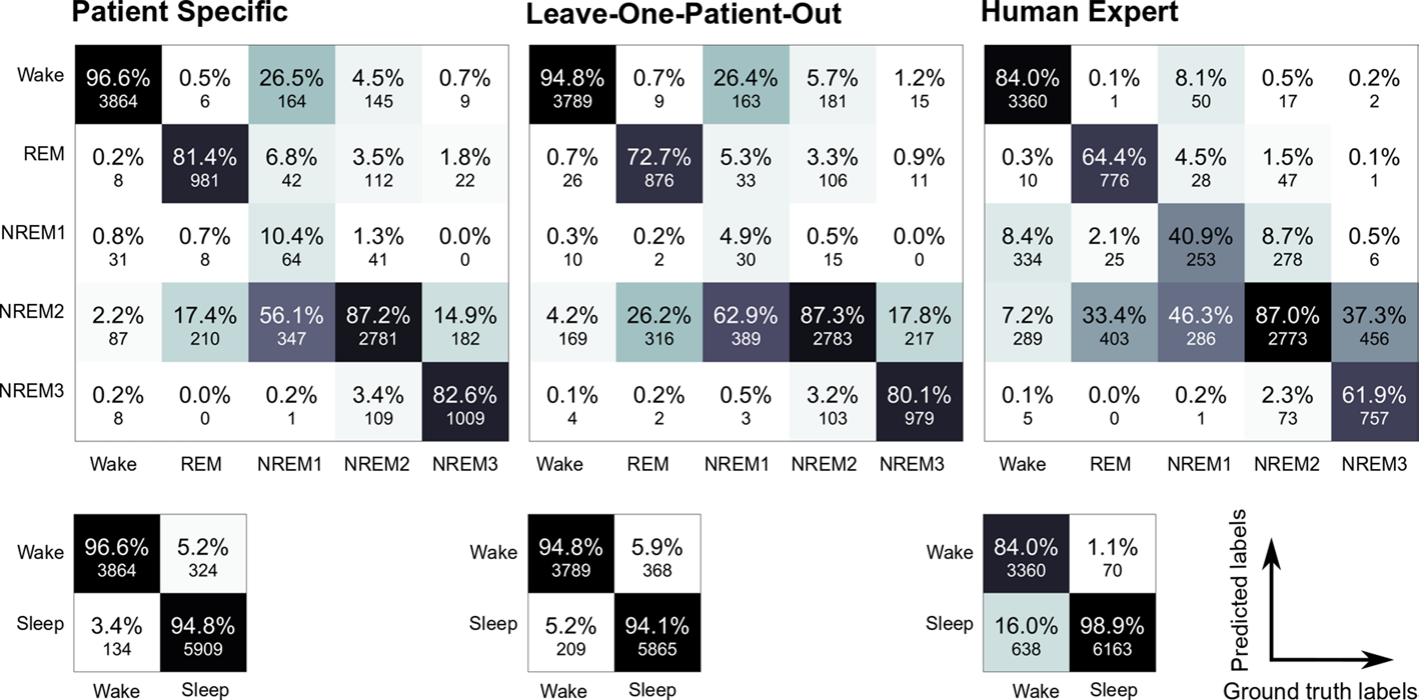
Confusion matrices for the five- and two-class problems. Each entry in the matrices provides the percentage *P* of epochs known to belong to class *i* that were classified as belonging to class *j*, for $i,j \in \{1, \ldots \text {NumberOfClasses}$, and the raw count. The percentage *P* in the diagonal equals the class sensitivity. The coloring reflects the magnitude of *P*, which ranges from 0 to 100 $\%$

The confusion matrices can be seen in Fig. 3. The PS algorithm classified 96.6% of the wake epochs correctly. The second best class sensitivity was seen for N2, where the individual approach classified 87.2% correctly. Then followed REM sleep with a class sensitivity of 81.4%, N3 with 82.6%, and lastly the N1 class with a poor class sensitivity of 10.4%. The order of the classes according to their class sensitivity was the same for the LONO approach and the human expert. However, the human expert had a substantially better performance on the N1 class, with a class sensitivity of 40.9%. On the simpler sleep–wake classification task, the performances were higher. The PS approach had a specificity of 96.6% and a sensitivity of 94.8%. The LONO approach performed similarly, with a specificity of 94.8% and a sensitivity of 94.1%. The human expert had a specificity of 84.0% and a sensitivity of 98.9%.

### Sleep measures

**Fig. 4:**
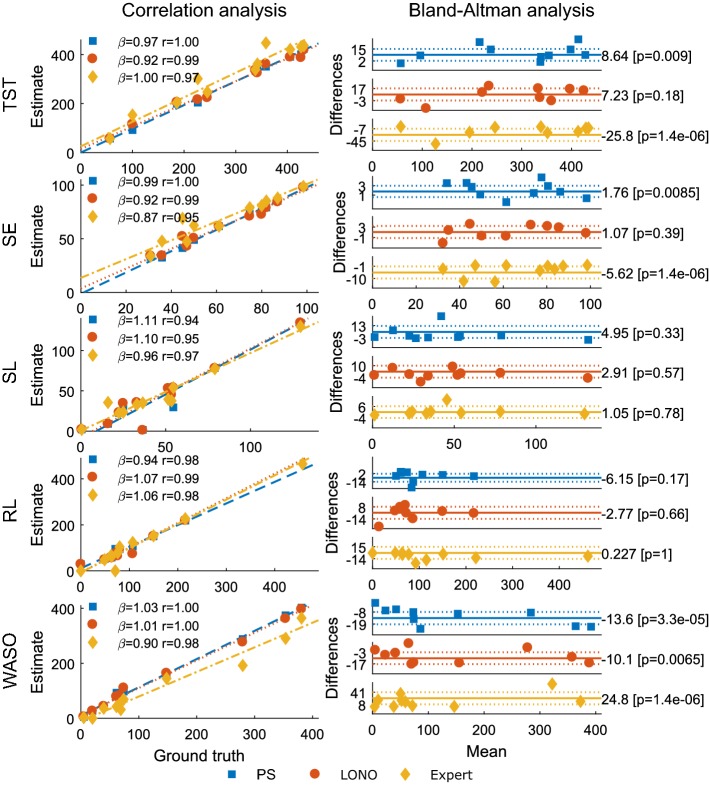
Comparison of ground truth sleep measures to estimated sleep measures. The blue squares indicate results from the PS algorithm, the red circles are the LONO algorithm and the yellow diamonds are the human expert. Left: scatter plot with Deming regression line, slope of regression line ($\beta$) and Pearson’s correlation coefficient (r). Right: Bland–Altman plots. The solid line is the mean difference, and the dotted lines are 1.96 times the standard deviation of the mean

A comparison of the estimated measures and the ground truth are shown Fig. 4. In general, there is a high agreement between the estimated and ground truth sleep measures as measured by the Deming slope and correlation coefficient, except for the estimation of REM latency by the PS algorithm. Here, the slope $\beta$ of the Deming regression line is 0.23 and the correlation coefficient $r=0.47$ due to a single outlier (patient D, night 3) where the ground truth latency is 457.5 min, and the estimated value is 100.5 min. As can be seen in Fig. 6, this night contains two sleep periods with a long wake period in between. According to the manually labeled hypnograms, the first REM epoch occurs after 457.5 min in the second sleep period. However, the PS algorithm predicts a single REM epoch after 100.5 min in the first sleep period.

The Bland–Altman analysis shows that there are three sleep measures where the difference in estimated and ground truth values are significant. The first is the TST, where the PS algorithm underestimates the measure by 8.64 min ($p=0.009$) and the human expert overestimates it by 25.8 min ($p<1\mathrm {e}{-4}$). The second measure is the SE, where the PS algorithm underestimates it by 1.76 min ($p=0.0085$) and the expert overestimates it by 5.62 min ($p<1\mathrm {e}{-4}$). The third is the WASO, where the PS algorithm overestimates it by 13.6 min ($p<1\mathrm {e}{-4}$), LONO overestimates it by 10.1 min ($p=0.0065$) and the expert underestimates it by 24.8 min ($p<1\mathrm {e}{-4}$).

## Discussion

We have automatically and manually sleep scored subcutaneous EEG from four patients with epilepsy, and achieved good results as compared to manually labeled LTV EEG. When classifying five stages, our best performing algorithm (the PS approach) achieved a mean Cohen’s kappa value of of 0.78 across patients, which according to McHugh et al. [18] represents a *moderate agreement*. When classifying sleep vs. wake, we achieved a mean kappa value of 0.85, which is in *strong* agreement.

The PS models outperformed the LONO models on almost all nights, even though the LONO models have more training data available in each CV fold. However, the majority of data in each training fold in the LONO approach originates from other patients than the one whose night is in the test fold. The difference in performance highlights the strength of long-term recording devices: the ability to provide large amounts of data from a specific person that can give rise to highly personalized algorithms rather than one-size-fits-all solutions.

The estimated sleep measures TST, SE, SL, RL and WASO were in good agreement with the ground truth values of the sleep measures computed from the manually labeled LTV EEG, as measured by the slope of the Deming regression line and correlation coefficient. Bland–Altman analysis revealed that there was a significant difference between the estimated values and the ground truth values for the TST, SE and WASO parameters across nights in the data set. However, the differences were relatively small for the PS models.

The algorithms outperformed the human expert on all performance measures except the class sensitivity of N1. However, this comparison is hardly fair, as the human expert had no training on subcutaneous EEG before scoring. There could possibly have been some learning for the human scorer during the course of analysis, but this was not tested for.

The result should be interpreted in the light of the weaknesses and strengths of this study. Firstly, it is based on a small data set with only four patients. However, multiple nights were recorded for each patient, allowing for training of patient-specific models with cross-validation schemes that is not based on random splitting epochs into train and test folds. By training on all nights except one and testing on the last night, the strong temporal inter-dependence between epochs from the same night is respected.

Secondly, the ground truth is manually scored hypnograms based on the full scalp EEG from LTV EEG, and not a PSG as according to the AASM guidelines. Although long-term PSG recordings would have been preferable, it was considered impractical and an unacceptable additional burden for the patients.

Thirdly, the placement of the subcutaneous electrodes was chosen to maximize the probability of recording temporal lobe seizures. This placement might be sub-optimal for sleep scoring, as some common sleep phenomena are best seen fronto-centrally [2]. As the implant can be placed at a variety of positions as long as the disk-shaped housing is located behind the ear, one might expect better results with a more optimal placement.

Finally, the 11 ground truth-hypnograms reflect how the patients slept poorly in the EMU. Furthermore, it is well established that epilepsy can induce sleep disturbances [19, 20]. The present classification performance might have improved if the data set consisted of 11 nights from four healthy subjects that were good sleepers.

As already mentioned, several relevant studies have sleep scored wearable EEG, and it is natural to compare these to the present study. Studies conducted on ear-EEG are perhaps the most relevant, as this modality also has the potential to provide unobtrusive, ultralong-term measurements. However, the hardware is still under development and there are no commercially available solutions yet. Nakamura et al. [13] conducted a small study on four healthy male subjects, where they recorded 45 min daytime naps after a sleep-deprived night using ear-EEG. They achieved a Cohen’s kappa of 0.65 when classifying the four stages W, N1, N2 and N3, and a kappa of 0.8 when classifying sleep vs. wake. Mikkelsen et al. [11] conducted a larger study using ear-EEG with nine healthy subjects. One night was recorded per subject. They achieved an average Cohen’s kappa value of 0.65 on five classes across subjects when training subject-specific models. By merging all sleep labels into a single sleep class, they achieved a sensitivity of 81% and a specificity of 97%. The present study shows better results, probably due to the fact that multiple nights were recorded per subject. In 2019, Mikkelsen et al. [12] did a larger study on 15 healthy subjects, this time using around-the-ear, flex-printed electrode arrays and Actiwatches (MW8, CamNtech, UK). Although the electrode arrays are less suitable for ultralong-term recordings, the experimental setup and aim of the study were similar to the present study. One night was recorded for each subject, and they used a leave-one-subject-out CV strategy. They achieved a mean Cohen’s kappa of 0.54 for the five-class problem and a mean Cohen’s kappa of 0.69 for the two-class problem. They found that the EEG-based device outperformed the Actiwatch in sleep detection. Mikkelsen et al. also estimated the five sleep measures TST, SE, SL, RL and WASO, and found that the estimates were in agreement with the true values except for RL and WASO. The significant underestimation of brief wake periods reported across several studies calls for future research on how to accurately estimate this parameter using wearable sleep monitors.

For sleep–wake detection, multiple studies have compared actigraphy to PSG. Most studies find that the sleep measures produced by the actigraphs are well correlated with the measures reported by PSG, but the modality often suffers from poor specificity. Kosmadopoulos et al. [21] assessed the validity of an Actiwatch-64 (Mini-Mitter Philips Respironics, Bend, OR) against PSG in 22 healthy subjects. By adjusting the activity threshold in the embedded sleep detection algorithm, they achieved sets of sensitivity and specificity ranging from 87.6 to 61.5 $\%$ (very low activity threshold) to 97.8% and 26.9% (high activity threshold). The Cohen’s kappa values ranged from 0.30 to 0.37. Slater et al. [22] assessed a GTX3+ Actigraph, and found a sensitivity, specificity and accuracy of 90, 46 and 84%, respectively. An extensive literature review on the subject was out of scope for this paper, but these results are in line with the review of Sadeh et al. [3]. Compared to actigraphy, EEG-based devices for ultralong-term sleep monitoring have two advantages: the ability to distinguish between sleep stages and a much higher specificity.

When developing and evaluating an alternative method, the question of ”how good is good enough” naturally arises. Surely, the goal of a medical device must be to obtain clinical relevance. Werner et al. [23] compared the sleep patterns of children as reported by actigraphy and sleep diaries. They compared several sleep measures, including TST and WASO. Based on the author’s clinical experience, the difference between the estimates based on actigraphy and sleep diary were considered in clinically acceptable agreement if it was less than 30 min. In the current study, the PS estimates of both TST and WASO as compared to the true values were within the 30 min limit for all nights. For the LONO approach, the estimates were all within the limit except for a single night.

When comparing Cohen’s kappa values, a reasonable benchmark for clinical relevance could be the inter-rater reliability (IRR) one might expect between different trained scorers. Danker-Hopfe et al. [24] found that the IRR as measured by Cohen’s kappa between scorers from eight European sleep laboratories was 0.6816. When the AASM guideline was introduced, the IRR increased to a kappa value of 0.76. As our best-performing algorithm achieved similar kappa values, we argue that the proposed method could deliver performances on par with current clinical practice.

As the data set only contained nights spent in the EMU, it remains an open question whether the algorithm can generalize to nights recorded in the patients’ own homes. Monitoring the sleep quality in patients with epilepsy over ultralong time periods could potentially have clinical value, as there is a complex interplay between sleep quality and epileptic activity. Studies have shown an improvement in seizure control when sleep disturbances were treated [20, 25]. Future research is needed to illuminate the clinical utility of ultralong-term EEG monitoring of sleep patterns in epilepsy patients recorded ”in the wild”.

## Conclusion

By recording several nights per patient, we were able to train patient-specific models and achieved a mean Cohen’s kappa value of 0.78 across recordings. This is higher than the inter-rater agreement one would expect between two human raters from different sleep laboratories, as reported by Danker-Hopf et al. When detecting sleep vs. wake, we achieved a sensitivity of 94.8% and a specificity of 96.6%, which is an improvement over the widely used actigraphy.

Of the five sleep measures TST, SE, SL, RL and WASO, we found significant differences in TST, SE and WASO. The differences were small and within reported clinically acceptable limits.

In conclusion, we are the first to show that sleep monitoring patients with epilepsy using subcutaneous EEG and automatic scoring algorithms is possible and can produce results of clinical relevance. Ultralong-term EEG combines the strengths of the PSG and actigraphy, providing both accurate sleep stage scoring and long-term measurements. With the possibility of recording ultralong measurements, there is a potential to develop strong patient-specific sleep scoring algorithms that could illuminate sleep pattern over weeks and months.

## Methods

### Data collection

Four adult epilepsy patients (one male, three female) with a temporally implanted two-channel EEG system were admitted for full channel workup in the Epilepsy Monitoring Unit (EMU) at Zealand University Hospital [2]. The four adults are a sub-population of a clinical study comprising nine adults with the implanted EEG system, but only four of the trial participants were admitted to the EMU [2]. During the patient’s EMU stay, LTV EEG and subcutaneous EEG were recorded simultaneously.

**Fig. 5:**
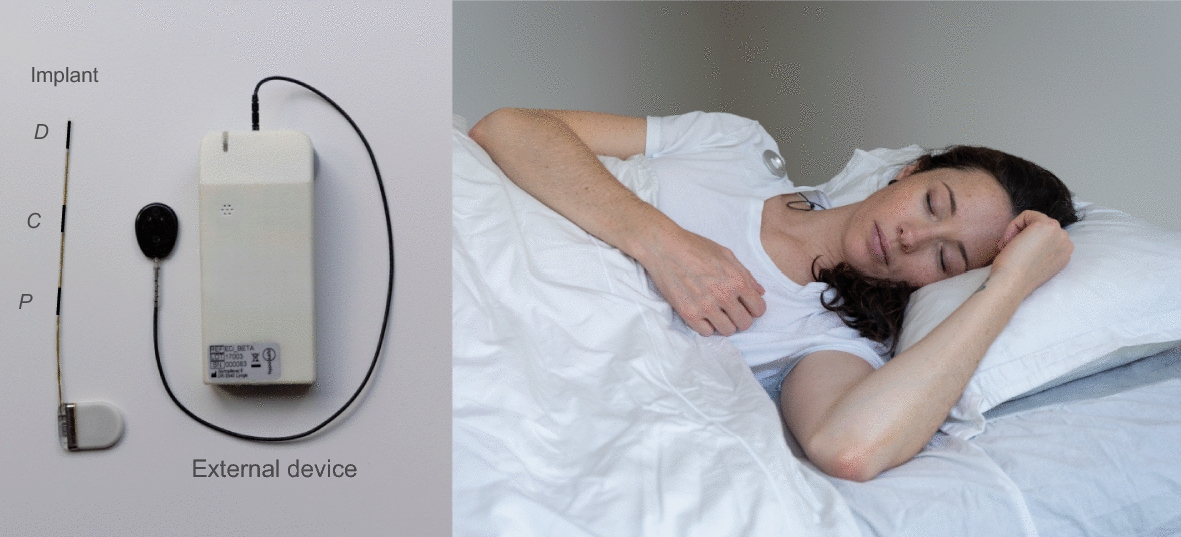
Illustration of the subcutaneous recordings system. Left: illustration of the implant and the beta-version of the external device used to collect data in the present study. The placement of the Proximal (P), Center (C) and Distal (D) electrodes are indicated by the letters. The length of the implant is approximately 11 cm. Right: illustration of the commercially available device. The device is worn under the shirt and secured in place by a magnet (gray circle)

The subcutaneous system consists of an implant and an externally worn device. The implant has three electrodes, where the center electrode acts as a reference to create two bipolar channels. It is implanted under the skin behind the ear under local anesthesia, and were placed such that the electrodes span the temporal lobe. The electrodes are named Distal (*D*), Center (*C*) and Proximal (*P*), where *P* is the closest to the ear. The external device contains a rechargeable battery and a memory chip. The external device both powers the implant and receives data through an inductive link across the skin, and has a sampling frequency of 207 Hz. The device is produced by UNEEG medical A/S (Lynge, Denmark), and in this study, a beta version of the *24/7 EEG SubQ* device was used. The commercially available device is marketed as a tool for treatment optimization by providing an objective estimation of the seizure burden. An illustration is provided in Fig. 5. The patients were given two external devices, one to wear during daytime (awake) and one for nighttime (sleep). The external devices were recharged when not in use. The time stamp for the start of the ”nighttime device”-recording is considered as ”lights off”. The LTV EEG was recorded with a NicoletOne wireless 64-channel head box (CareFusion 209) with a sampling rate of 1024 Hz. The 25 scalp EEG electrodes were placed according to the international 10-20 system with additional low row. The exact electrode placement can be seen in Appendix A.

The recordings from the two EEG modalities were sleep stage scored by a trained expert according to the AASM guidelines. The expert scored the recordings manually (and not computer assisted), as this is customary in Denmark. Only recordings from the period where the patient was wearing the subcutaneous ”night device” were considered, therefore any daytime naps are excluded. Both subcutaneous and scalp recordings were scored using Nicolet One version 5.95. For each patient, the subcutaneous recordings were scored before the scalp recordings, to get as unbiased subcutaneous scorings as possible.

### Data set

In total, 11 nights with concurrent scalp and subcutaneous EEG were recorded. Patient B had two nights, and the rest had three nights each. Patient A had two nights and patient C had one night where the external device was removed during the night. The part of the nights where both EEG modalities were recorded were included in the data set, as they were considered usable despite their short length. For an overview of age, gender, seizure onset zone, anti-epileptic drug intake, relevant MRI findings and total duration of EEG recordings for each EEG modality during the course of the EMU stay, the reader is referred to [2].

**Fig. 6:**
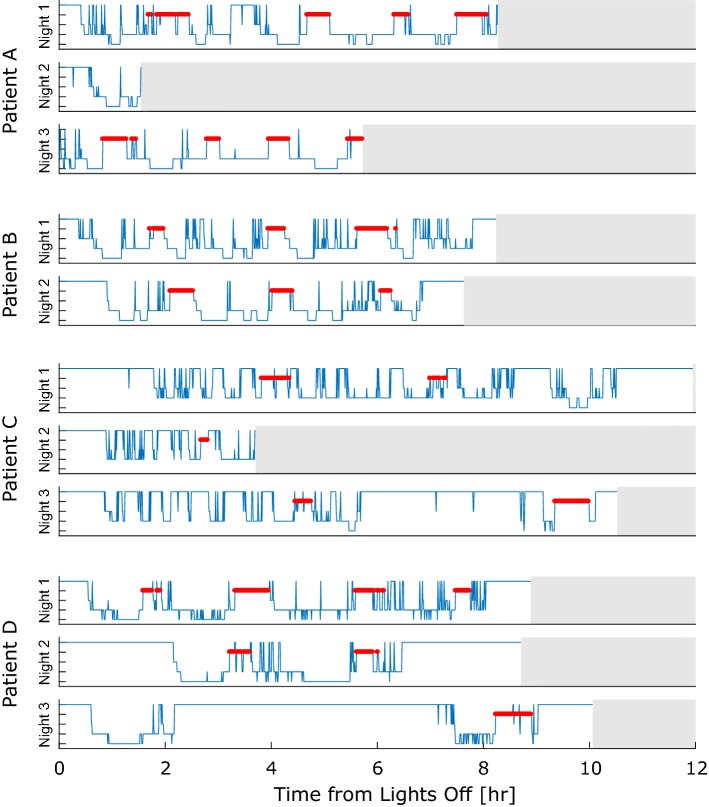
Manually scored hypnograms based on scalp EEG. The five tick marks on the y-axis represent (from top to bottom) wake, REM sleep, N1, N2 and N3. REM sleep is marked with a red, bold line. Three nights were recorded for each of patients A, C and D, and two nights were recorded for patient B

The hypnograms scored based on the LTV EEG, which are considered the ground truth, are visualized in Fig. 6. The hypnograms show that the nights are rather diverse. Patient D had trouble sleeping and patient C barely had any deep sleep (N3). The EEG technician at the EMU clinic noted that the patient most likely has an undiagnosed sleep apnea. Patient B had a seizure few hours before ”lights off” on both evenings, and patient D had >80 seizures during the EMU stay, the majority occurring in the evening before night 3. Observing poor sleep quality in the data set is therefore expected, as the extensive equipment setup, having epilepsy and the occurrence of seizures, can negatively impact sleep [11, 19].

### Data pre-processing and feature extraction

The EEG recordings and the hypnograms were imported into MATLAB version 2017a (MathWorks), in which all subsequent analysis was made. To synchronize the scalp and subcutaneous recordings, derivations with electrode placements similar to the subcutaneous placement were extracted from the scalp recordings. Both EEG recordings were band-pass filtered between 0.5 and 100 Hz, and notch filtered around 50 Hz. The scalp channels were downsampled to 207 Hz so that the EEG in the two recordings could be synchronized using the cross correlation. A crude artifact rejection was performed by removing parts of the EEG with an amplitude larger than 300 $\mu$V, to avoid having high amplitude artifacts dominate the cross correlation. The artifacts were removed from all subsequent EEG analysis. An overview of the amount of rejected artifact for each night can be seen in Appendix B. The synchronization was performed on a 30-s epoch-by-epoch basis, such that each scored epoch in the scalp EEG had a corresponding synchronized subcutaneous EEG epoch.

Thirty features were computed for each subcutaneous channel and epoch, and they are listed in Table 1. The features are based on the power distribution in the traditional frequency bands computed using MATLAB’s continuous wavelet transform (cwt). These features were chosen, as the AASM scoring rules to a large extent are based on activity in these frequency ranges. When computing the wavelet transform, the analytic Morse (3,60) wavelet was used, where 3 is the symmetry parameter and 60 is the time–bandwidth product. The traditional frequency bands were defined as delta: 0.5–4 Hz, theta: 4–8 Hz, alpha: 8–13, lower beta: 13-22 Hz and upper beta: 22–32 Hz. Note that the beta band has been divided into two separate bands, as it the traditional definition of this band is quite broad.

**Table 1:** Description of the 30 features that were computed for each EEG channel. The five frequency bands are the delta, theta, alpha, lower beta and upper beta

Feature number	Feature description
1–5	Mean power in the five frequency bands
6–10	Variance of the power distribution in the five frequency bands
11–15	Skewness of the power distribution in the five frequency bands
16–20	Kurtosis of the power distribution in the five frequency bands
21–25	Shannon entropy of the power distribution in the five frequency bands
26–30	Duration of the activation of the power in the five frequency bands

The five last features listed in the table are the duration of the activation of the power in the frequency bands. Here, ”activation” is defined as the mean power in a given frequency band exceeding a threshold of 1.5 times the median of the power in the range 0.5–100 Hz. As an example, the duration of the activation of the delta band for a given epoch is the amount of time that the mean power in the delta band exceeds the threshold during the 30 s epoch.

As the manual scoring of an epoch may depend on the label of the surrounding epochs, the epoched features were concatenated with the features from the preceding epoch and two subsequent epochs. Each epoch of EEG was thus represented by the features associated with four epochs.

#### Classification

By using the hypnogram from the scalp EEG as ground truth, an automatic sleep stage algorithm was trained on the features extracted from the synchronized two-channel subcutaneous EEG. The sleep staging algorithm used in this study is a random forest similar to [11, 12] that has scored around-the-ear-EEG with success. The forest consists of 100 decision trees fitted using the fitensemble function with the Bag method. The trees were trained by resampling the training data set with replacement. Every tree in the ensemble randomly selected features for node splitting. The tree nodes were split based on their impurity (Gini’s Diversity Index) using the standard CART algorithm. The splitting of a node continued until either the node was pure, there were fewer than ten observations in the node, or the algorithm had split $N_{TrainingSetSize}-1$ nodes. The model was cross-validated using two different schemes: patient specific (PS) and leave-one-night-out (LONO). In the PS scheme, one model was trained for each patient, in a leave-one-night-out manner. In the LONO approach, an 11-fold CV was achieved by using all possible combination of ten nights as training set, and using the remaining night as test set. Common for both CV strategies is that data samples from the same night are always in the same fold. This is in contrast to the often-used CV technique where epochs are assigned to training and test folds randomly, which ignores the strong dependence between epochs recorded close in time [5, 26].

Furthermore, we computed five sleep measures recommended by the AASM manual version 2.4 [27] from the hypnograms. The first measure is the total sleep time (TST), which is the total time spent asleep, and is the sum of the time spent in any of the sleep stages. Inspired by ANSI/CTA 2052.1 Standard *Definitions and Characteristics for Wearable Sleep Monitors* [28], we computed the time attempting to sleep (TATS) instead of the total recording time, which is used in the AASM manual. The TATS is a more suitable measure for continuous long-term recordings, and is defined as the time when the patient is in bed and starts attempting to sleep, until the patient is no longer attempting to sleep [28, 29]. TATS is in this study indicated by the mounting and dismounting of the nighttime subcutaneous EEG device. Sleep efficiency percentage (SE) is defined as TST/TATS $\times$ 100. Sleep latency (SL) is the time from when the patient begins attempting to sleep until the first sleep epoch of any stage occurs. Similarly, REM latency (RL) is defined as the time from first attempt to sleep to the first epoch of REM sleep. Wake after sleep onset (WASO) is defined as TATS–SL–TST. All sleep measures that are not given in percent are given in minutes. A set of ”ground truth sleep measures” was computed from the manually labeled hypnograms based on the scalp EEG and a set of ”estimated measures” was computed from the hypnograms predicted by the algorithms and the human expert based on the subcutaneous EEG.

In addition to computing hypnograms, we also derived sleep–wake traces, which is currently the standard in long-term sleep monitoring. The traces were derived by merging the sleep stages in the ground truth and predicted hypnograms post-analysis into a single sleep class. The algorithm was not retrained to do this, and the human expert did not re-score the same data.

### Evaluation

#### Sleep stage classification

To assess agreement between the predicted and ground truth hypnograms and sleep-wake traces, we computed Cohen’s kappa and confusion matrices. Cohen’s kappa coefficient ($\kappa$) is a statistic which measures interrater agreement for qualitative (categorical) items. It is generally thought to be a more robust measure than simple percent agreement calculation, as ($\kappa$) accounts for the possibility of the agreement occurring by chance. A kappa value was computed for each the 11 predicted hypnograms and sleep-wake traces for each method. A single confusion matrix was computed for each method. This corresponds to computing a population average weighted by the number of patient epochs. The confusion matrices report both the count and the percentage of epochs known to belong to class *i* that was classified as belonging to class *j* for $i,j \in \{1, \ldots , \text {NumberOfClasses} \}$. The percentage in the diagonal can be thought of as a class sensitivity. For the binary sleep detection problem, the first entry in the diagonal is the class sensitivity of the wake class. In the sleep literature however, it is customary to consider sleep epochs as positive data samples and wake epochs as negative samples. The first entry in the diagonal therefore equals the percentage of known wake epochs that were classified as such, TN/(TN+FP), which is often referred to as the specificity. Similarly, the second entry in the diagonal is the percentage of sleep epochs that were classified as sleep and is often referred to as the sensitivity.

#### Sleep measures

The ground truth and estimated values for the sleep measures were compared by means of scatter plots and Bland–Altman analysis. For the scatter plots, a straight line was fitted using Deming regression. Deming regression is suitable for data sets where both the X and Y variables are subject to measurement errors. If the sleep measures computed from the manually labeled hypnograms and the predicted hypnograms were in agreement, the slope of the Deming regression line should be close to 1. The Pearson correlation coefficient was also computed between the ground truth and estimated values.

Furthermore, a comparison between the ground truth and the estimated values were made using Bland–Altman analysis [30]. For each night and each sleep measure, the mean of the value of the ground truth sleep measure and the estimated sleep measure was computed, as well as the difference between the two values. The differences were plotted against their mean value, along with the mean difference value and its 95% confidence interval. A permutation test was performed on the differences to test whether the mean difference was different from zero [31]. A permutation test builds a reference distribution by resampling the observed data as opposed to assuming a reference distribution, as is done in a $\textit{t}$ test. As the number of samples here was low ($2\times 11$), it was feasible to run an exact test by considering all possible permutations of the samples when building the reference distribution. The significance level was set to 5%. Significant differences of positive sign implied that the proposed method was underestimating the value of the sleep measure as compared to the ground truth. Conversely, a negative mean difference implied that the proposed method overestimated the sleep measure.

## Data Availability

The datasets analyzed during the current study are not publicly available due to the interests of UNEEG^TM^ medical A/S. The code used to sleep score can be made available upon request.

## Appendix A

The scalp electrodes available for manual sleep scoring of the long-term video EEG are: Fp1, Fp2, F3, F4, C3, C4, P3, P4, ,O1, O2, F7, F8, T7, T8, P7, P8, Fz, Cz, Pz, F9, F10, T9, T10, P9, and P10.

## Appendix B

A crude artifact rejection was performed on both EEG modalities before synchronization. After synchronization, the parts that were excluded from one of the modalities, were also excluded from the other, in order to make sure that both modalities had the same amount of data.

**Table:**

Patient	Night	Rejected data (min)
A	1	104
	2	41.9
	3	45.3
B	1	3.28
	2	13.7
C	1	40.6
	2	10.3
	3	11.5
D	1	11.6
	2	21.1
	3	40.3

## Appendix C

List of Cohen’s kappa values for the sleep stage classification and the sleep-wake classification. See Tables 2, 3.

**Table 2:** Cohen’s kappa values for the 5-class problem

Patient	Night	PS $\kappa$	LONO $\kappa$	Expert $\kappa$
A	1	0.81	0.79	0.80
	2	0.84	0.83	0.80
	3	0.84	0.83	0.90
B	1	0.62	0.56	0.56
	2	0.82	0.71	0.56
C	1	0.71	0.69	0.59
	2	0.82	0.67	0.43
	3	0.78	0.77	0.59
D	1	0.73	0.70	0.60
	2	0.79	0.77	0.73
	3	0.79	0.77	0.74
Mean (± SD)		0.78 (± 0.02)	0.74 (± 0.02)	0.66 (± 0.04)

**Table 3:** Cohen’s kappa values for the 2-class problem

	Night	PS $\kappa$	LONO $\kappa$	Expert $\kappa$
A	1	0.81	0.79	0.83
	2	0.92	0.92	0.94
	3	0.58	0.53	0.70
B	1	0.84	0.83	0.81
	2	0.88	0.85	0.87
C	1	0.90	0.85	0.74
	2	0.89	0.76	0.49
	3	0.91	0.92	0.73
D	1	0.82	0.78	0.88
	2	0.91	0.90	0.97
	3	0.92	0.90	0.92
Mean (± SD)		0.85 (± 0.03)	0.82 (± 0.03)	0.81 (± 0.04)

## Abbreviations

AASM: American Academy of Sleep Medicine
CART: Classification And Regression Tree
CV: cross-validation
EEG: electroencephalography
EMU: epilepsy monitoring unit
IRR: inter-rater reliability
LONO: leave-one-night-out
LTV EEG: long-term video-EEG
MRI: magnetic resonance imaging
N1: non-rapid eye movement stage 1
N2: non-rapid eye movement stage 2
N3: non-rapid eye movement stage 3
PS: patient specific
PSG: polysomnography
REM: rapid eye movement
RL: rapid eye movement-latency
SE: sleep efficiency
SL: sleep latency
TATS: time attempting to sleep
TST: total sleep time
WASO: wakefulness after sleep onset
